# Retreatment With EGFR-Tyrosine Kinase Inhibitor After Disease Progression Following Gefitinib Induction and Chemoradiotherapy in EGFR-Mutant Stage III Non-small Lung Cancer: An Efficacy and Safety Analysis of the LOGIK0902/OLCSG0905 Study

**DOI:** 10.7759/cureus.86575

**Published:** 2025-06-23

**Authors:** Sho Saeki, Katsuyuki Hotta, Shinya Sakata, Naohiro Oda, Koji Inoue, Tomoki Tamura, Ryo Toyozawa, Daijiro Harada, Kentaro Tanaka, Koji Inoue, Yoshiyuki Shioyama, Kenichi Gemba, Tomonari Sasaki, Akihiro Bessho, Junji Kishimoto, Kuniaki Katsui, Katsuyuki Kiura, Kenji Sugio

**Affiliations:** 1 Department of Respiratory Medicine, Kumamoto University Hospital, Kumamoto, JPN; 2 Center for Innovative Clinical Medicine, Okayama University Hospital, Okayama, JPN; 3 Internal Medicine, Fukuyama City Hospital, Fukuyama, JPN; 4 Department of Respiratory Medicine, Okayama University Hospital, Okayama, JPN; 5 Department of Respiratory Medicine, Kitakyushu Municipal Medical Center, Kitakyushu, JPN; 6 Department of Respiratory Medicine, National Hospital Organization Iwakuni Clinical Center, Iwakuni, JPN; 7 Department of Thoracic Oncology, National Hospital Organization Kyushu Cancer Center, Fukuoka, JPN; 8 Department of Thoracic Oncology, National Hospital Organization Shikoku Cancer Center, Matsuyama, JPN; 9 Department of Respiratory Medicine, Graduate School of Medical Sciences, Kyushu University, Fukuoka, JPN; 10 Department of Respiratory Medicine, Ehime Prefectural Central Hospital, Matsuyama, JPN; 11 Radiation Oncology, Ion Beam Therapy Center, SAGA HIMAT Foundation, Tosu, JPN; 12 Department of Respiratory Medicine, Chugoku Central Hospital, Fukuyama, JPN; 13 Department of Radiation Oncology, Iizuka Hospital, Iizuka, JPN; 14 Department of Respiratory Medicine, Japanese Red Cross Okayama Hospital, Okayama, JPN; 15 Center for Clinical and Translational Research, Kyushu University Hospital, Fukuoka, JPN; 16 Department of Radiology, Division of Radiation Oncology, Kawasaki Medical School, Kurashiki, JPN; 17 Thoracic and Breast Surgery, Oita University, Yufu, JPN

**Keywords:** chemoradiotherapy, egfr, locally advanced setting, non-small cell lung cancer, progression, retreatment, safety, targeted therapy

## Abstract

Background and objective

We had previously conducted a phase II study (LOGIK0902/OLCSG0905 study) involving the eight-week administration of gefitinib, followed by cisplatin-based chemoradiotherapy, to treat locally advanced, epidermal growth factor receptor (EGFR)-mutated, non-small cell lung cancer (NSCLC). Despite favorable overall survival outcomes, more than half of the patients relapsed after the protocol therapy, highlighting the need to clarify the clinical significance of retreatment with EGFR-tyrosine kinase inhibitors (TKIs). We investigated the efficacy and safety of EGFR-TKI retreatment after disease progression.

Materials and methods

We included 14 patients who relapsed after the protocol treatment and received any type of EGFR-TKI as post-progression treatment in this sub-analysis. We evaluated the efficacy and safety of retreatment with EGFR-TKI in these patients.

Results

Among the 14 patients, 11 (78.6%) responded to the induction of gefitinib in the treatment protocol. After relapse, 9/14 patients (64.3%) received gefitinib, 3/14 (21.4%) received afatinib, and 2/14 (14.3%) received erlotinib monotherapy, respectively. The median duration of post-progression EGFR-TKI treatment was 17.9 (0.7-45.5) months. The overall response rate (ORR) and disease control rate were 64.3% [9/14 patients; 95% confidence interval (CI): 35.1%-87.2%] and 85.7% (12/14 patients; 95% CI: 57.2%-98.2%), respectively. The median progression-free survival (PFS) and median survival durations after the initiation of EGFR-TKI retreatment were 11.8 months (95% CI: 5.7-20.7 months) and 47.4 months (95% CI: 31.8 months to not estimable), respectively. Adverse events were comparable to those previously reported.

Conclusions

Patients with disease progression after protocol therapy demonstrated sensitivity to retreatment with an EGFR-TKI, with acceptable safety.

## Introduction

The current standard of care for patients with unresectable locally advanced non-small cell lung cancer (LA-NSCLC) includes the addition of consolidative durvalumab after the completion of concurrent chemoradiotherapy [1,2]. The results of the LAURA trial involving patients with unresectable stage III epidermal growth factor receptor (EGFR)-mutant NSCLC were recently published. The LAURA trial evaluated the effects of osimertinib administered until disease progression in patients who did not show progression after standard chemoradiotherapy. Progression-free survival (PFS), the primary endpoint, was significantly prolonged in the osimertinib group (39.1 months) compared with that in the placebo group (5.6 months) [hazard ratio (HR): 0.16; 95% confidence interval (CI): 0.10-0.24; p<0.001) [3]. As the LAURA trial had a median follow-up period of less than two years, caution should be exercised when interpreting overall survival (OS) data. Furthermore, it is still unclear when the best timing to administer an EGFR-tyrosine kinase inhibitor (TKI) for EGFR-mutant, locally advanced tumors is in order to achieve favorable treatment outcomes.

We conducted the first prospective phase II study on gefitinib induction followed by chemoradiotherapy in 20 patients with EGFR-mutant LA-NSCLC [4,5]. The protocol therapy yielded promising results with a two-year survival rate of 90% (18/20 patients; 90% CI: 71.4-96.8) and an overall response rate (ORR) of 85% (17/20 patients). However, 15/20 patients (75%) eventually experienced recurrence during the median follow-up period of 47.5 months, implying the potential effect of treatments after progression on post-progression survival. There have been no studies on the efficacy and safety of retreatment with EGFR-TKIs in this setting. Therefore, in this study, we aimed to investigate the clinical effect of EGFR-TKI retreatment after disease progression in the LOGIK0902/OSCLG0905 trial.

## Materials and methods

Study design

The LOGIK0902/OLCSG0905 study involved patients with inoperable LA-NSCLC harboring EGFR mutations (exons 19 or 21). These patients underwent an eight-week induction with gefitinib monotherapy, followed by standard chemoradiotherapy. The eligibility criteria and protocol of treatment had been reported previously [4,5]. Briefly, the study involved patients with a median age of 74 years, an Eastern Cooperative Oncology Group (ECOG) performance status of 0-1, and pathologically confirmed unresectable stage IIIA/IIIB disease (7th TNM classification and staging system) with EGFR mutations in exon 19 or 21. Imaging assessments for disease progression were performed using CT at least every three months and brain MRI every three to six months. Post-progression treatment, including the type of EGFR-TKI, cytotoxic chemotherapy, and radiotherapy, was not defined specifically in the protocol; it was determined by the physicians in charge of each participant. For this study, we enrolled patients between April 2011 and January 2017; the study was conducted before the approval of third-generation EGFR-TKIs.

In this sub-analysis, we assessed patients who experienced disease progression after the LOGOL0902/OLCSG0905 protocol treatment and received any additional EGFR-TKI post-progression treatment. Patients who received EGFR-TKIs before disease progression, as confirmed after treatment with the LOGOL0902/OLCSG0905 protocol, were excluded from this study. If EGFR-TKIs were used in two or more lines of therapy after the first recurrence, clinical data regarding the earliest treatment line used as post-progression therapy were reviewed. The data cutoff was set at February 28, 2021. The study protocol was approved by the institutional review board and independent ethics committees: Okayama University Hospital Ethics Committee (approval number: rin1045; date: January 25, 2011) and the Clinical Research Network Fukuoka Certified Review Board (approval number: 18-C24; date: February 20, 2019). Written informed consent was obtained from all the patients.

Outcomes and statistical analysis

The response to EGFR-TKI as a post-treatment was evaluated radiographically according to the Response Evaluation Criteria in Solid Tumors version 1.1. Adverse events were assessed according to the National Cancer Institute Common Terminology Criteria for Adverse Events, version 4.0. PFS was defined as the time from the date of restarting EGFR-TKI after recurrence on the original protocol therapy to the date of confirmation of progressive disease or date of death from any cause. OS was defined as the time to death from any cause. PFS and OS were analyzed using the Kaplan-Meier method. SAS version 9.4 (SAS Institute, Cary, NC) was used for all analyses.

## Results

Patients and recurrence patterns 

Among the 15 patients who experienced disease progression after protocol treatment, one was excluded from this sub-analysis because the protocol treatment (gefitinib induction) was discontinued due to hepatotoxicity, and the subsequent EGFR-TKI therapy was initiated before disease progression. All the other patients received retreatment with a single-agent EGFR-TKI after the first event of disease progression and were included in the sub-analysis. The baseline characteristics of the 14 patients are summarized in Table 1. There were 10 female patients (10/14; 71.4%); the median age of the patients was 67.5 years, and eight patients (8/14; 57.1%) had point mutations in EGFR exon 21. The most common site of progression was the brain (6/14; 42.9%). Nine (9/14; 64.3%) patients received gefitinib, three (3/14; 21.4%) received afatinib, and two (2/14; 14.3%) received erlotinib monotherapy as post-progression EGFR-TKI treatment. The ORR and median PFS duration for gefitinib in the protocol treatment were 78.6% (11/14; 95% CI: 57.1%-100%) and 11.8 (95% CI: 7.6-20.9) months, respectively.

**Table 1:** Baseline characteristics before the initial EGFR-TKI administration after disease progression (n=14) CI: confidence interval; EGFR: epidermal growth factor receptor; ORR: overall response rate; PFS: progression-free survival; PS: performance status; TKI: tyrosine kinase inhibitor

Characteristics		Values
			Sex, n (%)	Male	4 (28.6)
				Female	10 (71.4)
Age, years	Median (range)	67.5 (55–74)
PS, n (%)	0	5 (35.7)
	1	9 (64.3)
Type of EGFR mutation, n (%)	Exon 21, L858R	8 (57.1)
	Exon 19 deletions	6 (42.9)
Site of progression, n (%)	Brain	6 (42.9)
	Lung	4 (28.6)
	Lymph node	1 (7.1)
	Adrenal gland	1 (7.1)
	Liver	1 (7.1)
	Pleura	1 (7.1)
EGFR-TKI, n (%)	Gefitinib	9 (64.3)
	Erlotinib	2 (14.3)
	Afatinib	3 (21.4)
Radiotherapy before EGFR-TKI, n (%)	None	9 (64.3)
	Primary lesion	1 (7.1)
	Brain	4 (28.6)
Efficacy of gefitinib in the protocol treatment	ORR, % (95% CI)	78.6 (57.1–100)
	Median PFS, months (95% CI)	11.8 (7.6–20.9)

Post-progression EGFR-TKI therapy

**Figure 1:**
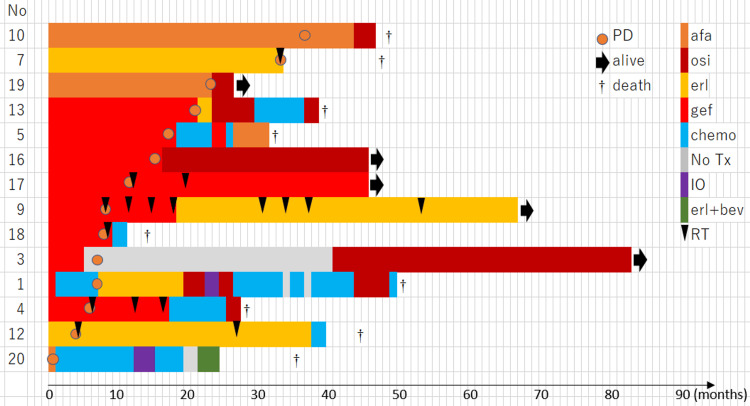
Swimmer plot of overall survival for retreatment with EGFR-TKI Each plot is drawn from the date of restarting EGFR-TKI therapy following recurrence after the original protocol therapy to the date of death from any cause afa: afatinib; chemo: chemotherapy; EGFR: epidermal growth factor receptor; erl: erlotinib; erl+bev: erlotinib and bevacizumab; gef: gefitinib; IO: immuno-oncology drug; No Tx: supportive care alone without any anti-cancer treatment; osi: osimertinib; PD: progressive disease; TKI: tyrosine kinase inhibitor

The median duration of post-progression EGFR-TKI treatment was 17.9 (0.7-45.5) months for all 14 patients, and one patient (1/14; 7.1%) was receiving EGFR-TKI treatment at the time of data cutoff. Dose reduction was required for four (4/14; 28.6%) patients, whereas treatment interruption was required for eight (8/14; 57.1%) patients. Swimmer plots illustrating the reinitiation of EGFR-TKI treatment are shown in Figure 1.

Efficacy of post-progression EGFR-TKI treatment

The ORR and disease control rate were 64.3% (9/14 patients; 95% CI: 35.1%-87.2%) and 85.7% (12/14 patients; 95% CI: 57.2%-98.2%), respectively (Table 2).

**Table 2:** Tumor response to EGFR-TKI retreatment after disease progression in the LOGIK0902/OLCSG0905 study (n=14) CI: confidence interval; EGFR: epidermal growth factor receptor; TKI: tyrosine kinase inhibitor

Variables	Values	
Overall best response, n (%)		
Complete response	0 (0)	
Partial response	9 (64.3)	
Stable disease	3 (21.4)	
Progressive disease	1 (7.1)	
Not evaluated	1	
Objective response rate, % (95% CI)	64.3 (35.1–87.2)	
			Disease control rate, % (95% CI)	85.7 (57.2–98.2)	

**Figure 2:**
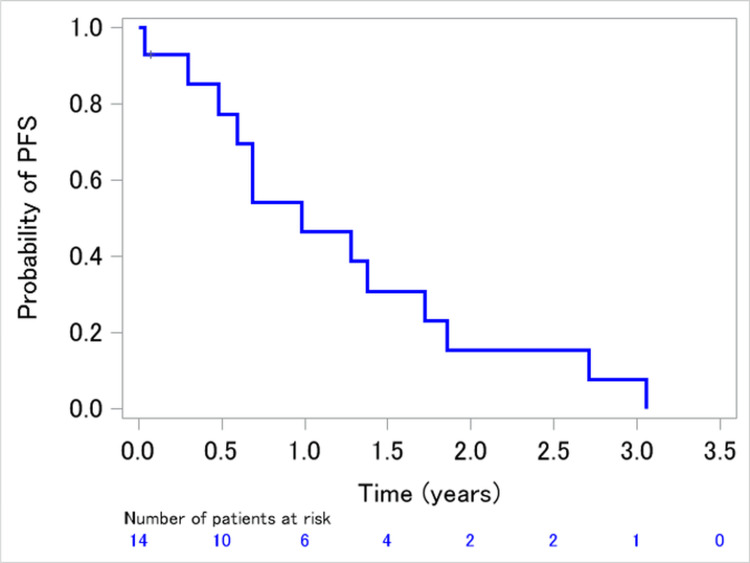
Kaplan–Meier survival curve showing PFS of patients retreated with an EGFR-TKI EGFR: epidermal growth factor receptor; PFS: progression-free survival; TKI: tyrosine kinase inhibitor

**Figure 3:**
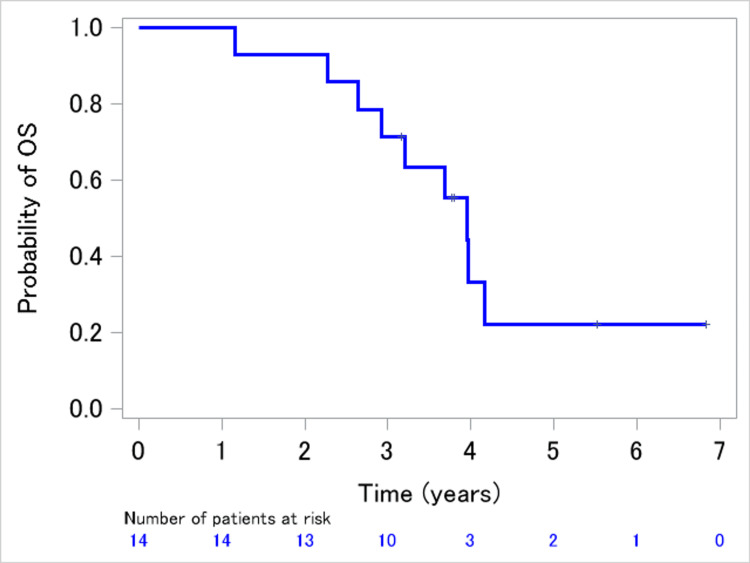
Kaplan–Meier survival curve showing OS of patients retreated with an EGFR-TKI EGFR: epidermal growth factor receptor; OS: overall survival; TKI: tyrosine kinase inhibitor

The median PFS and median survival durations were 11.8 months (95% CI: 5.7-20.7 months; Figure 2) and 47.8 months (95% CI: 31.8 months to not estimable; Figure 3), respectively.

A comparison of the ORR, PFS, and OS according to clinical factors is shown in Table 3; no clinically significant differences were observed. 

**Table 3:** Efficacy of EGFR-TKI retreatment stratified by clinical factors Cutoff based on the median score AE: adverse events; CI: confidence interval; EGFR: epidermal growth factor receptor; NE: not estimable; ORR: overall response rate; PFS: progression-free survival; PS: performance status; TKI: tyrosine kinase inhibitor

Factor	Category	N	Median PFS (months)	95% CI	Median OS (months)	95% CI	ORR (%)	95% CI
Overall		14	11.8	5.7–20.7	47.4	31.8–NE	64.3	(35.1–87.2)
Age, years	<65	5	15.3	0.4–22.3	35.2	27.2–NE	80	(28.4–99.5)
	≥65	9	10	3.6–32.6	47.5	13.8–NE	55.6	(21.2–86.3)
Sex	Male	4	8.2	3.6–32.6	45.8	13.8–49.9	50	(6.8–93.2)
	Female	10	13.6	0.4–20.7	47.5	27.2–NE	70	(34.8–93.3)
PS	0	5	12.4	8.2–22.3	49.9	13.8–NE	60	(14.7–94.7)
	1	9	11.8	0.4–32.6	47.4	27.2–NE	66.7	(29.9–92.5)
Smoking history	Never	9	15.3	0.4–22.3	47.5	31.8–NE	66.7	(29.9–92.5)
	Ever	5	7	3.6–32.6	44.2	13.8–49.9	60	(14.7–94.7)
Type of EGFR mutation	Exon 19	6	11.8	0.4–20.7	47.1	38.4–NE	66.7	(22.3–95.7)
	Exon 21	8	12.4	5.7–32.6	47.4	13.8–NE	62.5	(24.5–91.5)
PFS in protocol therapy, months	≤11.8	7	11.8	0.4–20.7	47.4	35.2–49.9	57.1	(18.4–90.1)
	>11.8	7	11.8	3.6–36.6	47.5	13.8–NE	71.4	(29.0–96.3)
Induction therapy	No response	3	20.7	0.4–36.6	38.4	35.2–47.5	33.3	(0.8–90.6)
	Response	11	10	3.6–16.5	47.4	27.2–NE	72.7	(39.0–94.0)
Chemoradiotherapy	No response	8	15.9	3.6–32.6	47.5	31.8–NE	62.5	(24.5–91.5)
	Response	5	11.8	0.4–20.7	38.4	13.8–49.9	60	(14.7–94.7)
AE grade ≥3	No	6	13.6	7.2–32.6	47.4	13.8–NE	83.3	(35.9–99.6)
	Yes	8	8.2	0.4–22.3	44.2	27.2–49.9	50	(15.7–84.3)

Re-biopsy before and after retreatment with an EGFR-TKI

Three patients underwent re-biopsy before retreatment with EGFR-TKIs: a biopsy of the primary lesion was performed for one patient, and biopsies of lung metastases were performed for two patients. After retreatment with EGFR-TKIs, 13 patients experienced progression, 12/13 (85.7%) of whom underwent re-biopsy at that time; the tumor biopsy sites were the lung, lymph node, bone, and pleura in 2/14 (14.3%) patients each, and liver in 1/14 (6.7%). Liquid biopsy from plasma was performed in 3/14 (21.4%) patients. The re-biopsy revealed T790M-positivity in 5/12 (41.7%) patients.

Toxicity

The toxicity profiles are shown in Table 4. Although aspartate aminotransferase (7/14; 50%) and alanine aminotransferase level elevations (8/14; 57%) were common, grade 3 events, including treatment-related deaths (0/14), were not observed. Skin toxicity was frequent, including acneiform eruption (12/14; 85.7%) and paronychia (4/14; 28.6%), but was generally mild and comparable to that reported previously. No pneumonia events were observed. One patient developed an acute myocardial infarction but recovered with appropriate treatment (including percutaneous coronary intervention); this event was considered unlikely to be related to retreatment with EGFR-TKIs.

**Table 4:** Toxicities recorded in the study (n=14)

Variables	Any grade, n (%)	Grade ≥3, n (%)
Leukopenia	6 (43%)	0
Neutropenia	4 (29%)	0
Anemia	8 (57%)	1 (7%)
Thrombocytopenia	7 (50%)	0
Aspartate aminotransferase level elevation	7 (50%)	0
Alanine aminotransferase level elevation	8 (57%)	0
Creatinine level elevation	3 (21%)	0
Hyperbilirubinemia	1 (7%)	0
Fatigue	1 (7%)	1 (7%)
Appetite loss	2 (14%)	0
Diarrhea	7 (50%)	0
Acneiform eruption	12 (86%)	2 (14%)
Oral mucositis	3 (21%)	0
Paronychia	4 (29%)	1 (7%)
Pneumonitis	0	0
Acute myocardial infarction	1 (7%)	1 (7%)

## Discussion

Our study demonstrated that retreatment with EGFR-TKIs had a favorable response rate, even as post-progression therapy after the initial eight-week gefitinib treatment and chemoradiotherapy. Response rates for first- and second-generation EGFR-TKIs as first-line treatments have been reported to be 60-80%, with PFS duration of 9-13 months [6-11]. Furthermore, in a retrospective analysis, the median PFS duration for EGFR-TKI treatment was reported to be 8.3 months (95% CI: 5.5-14.8 months) in 21 patients with EGFR-mutant, EGFR-TKI-naive LA-NSCLC who relapsed after the standard chemoradiation [12]. The efficacy of the retreatment in our study is comparable to or superior to that in these previous studies [6-12]. The LOGIK0902/OLCSG0905 study aimed to improve cure rates, but pretreatment with EGFR-TKIs raised concerns about reduced sensitivity after relapse. A possible explanation for the maintained sensitivity to EGFR-TKIs in this study could be that eight weeks of induction therapy with EGFR-TKIs was too short for the development of resistance. Another possibility is that chemotherapy and radiotherapy may have eliminated resistant clones that could be resistant to EGFR-TKIs and restored sensitivity.

Kanda et al. conducted a prospective phase II trial of gefitinib with intermittent introduction of other chemotherapeutic agents in patients with advanced NSCLC harboring EGFR mutations [13]. The study design involved the introduction of three cycles of cisplatin and docetaxel after eight weeks of gefitinib administration, followed by retreatment with gefitinib; the reported median PFS duration of 19.5 months and median OS duration of 48.0 months suggest that the treatment approach prevented the development of acquired resistance to EGFR-TKIs. Another phase II study evaluated gefitinib as a third-line treatment after first-line gefitinib and second-line chemotherapy in patients with advanced NSCLC harboring EGFR mutations. The study reported a median PFS duration of 4.4 months and a median OS duration of 10.3 months, suggesting that second-line chemotherapy may restore sensitivity to EGFR-TKI [14]. Taken together, our results suggest that eight weeks of induction therapy with EGFR-TKIs does not attenuate the efficacy of EGFR-TKI treatment after relapse, and that chemoradiotherapy may reduce the proportion of resistant clones, thereby enhancing the efficacy of subsequent EGFR-TKI therapy.

Regarding safety, similar to the findings of the original study, there was no increase in the incidence of pneumonitis. The incidence of common adverse events, such as skin toxicity and liver dysfunction, did not increase with retreatment with EGFR-TKIs, and no new adverse events were observed. Re-biopsy after retreatment with EGFR-TKI was performed at various sites, including the lung, lymph node, bone, pleura, liver, and plasma. The positivity rate of the T790M mutation was comparable to that in previous studies on first- and second-generation EGFR-TKI [15,16]. This finding suggests that induction therapy with EGFR-TKIs does not affect the resistance mechanism after subsequent EGFR-TKI retreatment.

Limitations

This study has some limitations. Firstly, it was a sub-analysis of a study with a small cohort; hence, findings should be interpreted with caution. Second, treatment decisions after disease progression were not standardized or randomized but rather left to the discretion of the treating physician. In addition, the protocol did not specify detailed methods for evaluating efficacy and adverse events, and no formal power calculation or sample size rationale was provided, which may affect the reproducibility and generalizability of the findings. Finally, we reported findings primarily on the use of first- and second-generation EGFR-TKIs; it remains unclear whether third-generation EGFR-TKIs are safe and effective for retreatment in this setting.

## Conclusions

This study suggests that post-progression retreatment with EGFR-TKIs is both effective and safe in patients with locally advanced non-small cell lung cancer harboring EGFR mutations who experienced disease progression after initial gefitinib induction and chemoradiotherapy. These findings may inform future treatment strategies for EGFR mutation-positive LA-NSCLC.
